# The interleukin 1 inhibitor rilonacept in treatment of chronic gouty arthritis: results of a placebo-controlled, monosequence crossover, non-randomised, single-blind pilot study

**DOI:** 10.1136/ard.2009.108936

**Published:** 2009-07-26

**Authors:** R Terkeltaub, J S Sundy, H R Schumacher, F Murphy, S Bookbinder, S Biedermann, R Wu, S Mellis, A Radin

**Affiliations:** 1 VAMC/UCSD, San Diego, USA; 2 Rheumatology and Immunology, Duke University Medical Center, Durham, USA; 3 University of Pennsylvania, VAMC, Philadelphia, USA; 4 Altoona Center for Clinical Research, Duncansville, USA; 5 Ocala Rheumatology Research Center, Ocala, USA; 6 Translational Medicine, Regeneron Pharmaceuticals Inc, Tarrytown, USA

## Abstract

**Background:**

Recent studies suggest that blockade of the NLRP3 (cryopyrin) inflammasome interleukin 1β (IL1β) pathway may offer a new treatment strategy for gout.

**Objective:**

To explore the potential utility of rilonacept (IL1 Trap) in patients with chronic active gouty arthritis in a proof-of-concept study.

**Methods:**

This 14-week, multicentre, non-randomised, single-blind, monosequence crossover study of 10 patients with chronic active gouty arthritis included a placebo run-in (2 weeks), active rilonacept treatment (6 weeks) and a 6-week post-treatment follow-up.

**Results:**

Rilonacept was generally well tolerated. No deaths and no serious adverse events occurred during the study. One patient withdrew owing to an injection-site reaction. Patients’ self-reported median pain visual analogue scale scores significantly decreased from week 2 (after the placebo run-in) to week 4 (2 weeks of rilonacept) (5.0 to 2.8; p<0.049), with sustained improvement at week 8 (1.3; p<0.049); 5 of 10 patients reported at least a 75% improvement. Median symptom-adjusted and severity-adjusted joint scores were significantly decreased. High-sensitivity C-reactive protein levels fell significantly.

**Conclusions:**

This proof-of-concept study demonstrated that rilonacept is generally well tolerated and may offer therapeutic benefit in reducing pain in patients with chronic refractory gouty arthritis, supporting the need for larger, randomised, controlled studies of IL1 antagonism such as with rilonacept for this clinical indication.

A growing subpopulation of patients with gout can be characterised as having “difficult gout”.^1^ These patients are often intolerant of, or refractory to, standard therapeutic approaches to gouty inflammation—non-steroidal anti-inflammatory drugs (NSAIDs), systemic or intra-articular glucocorticosteroids, or colchicine.^2^ Many patients with difficult gout have demonstrated intolerance to, contraindications to, or failure of multiple available urate-lowering treatments.^1^ ^3^ ^4^ ^5^

Recent data support an important role for interleukin 1β (IL1β) in the inflammatory process associated with monosodium urate (MSU) crystal deposits in tissues of patients with gout. MSU crystals promote inflammation in large part by inducing activation of the cryopyrin (NLRP3) inflammasome, an intracellular, multiprotein complex responsible for cleavage of caspase-1 that is essential for the processing and secretion of IL1β.^6^ The release of IL1β^7^ ^8^ promotes neutrophil influx into the joint that both drives and sustains gouty inflammation.^1^ A recent, open-label, uncontrolled pilot study of the soluble IL1 receptor antagonist anakinra suggested benefit in refractory human gouty inflammation.^9^

Rilonacept, a soluble receptor-Fc fusion protein, engages and inhibits both IL1α and IL1β and has demonstrated rapid and durable effects in a phase 3 study of patients with cryopyrin-associated periodic syndromes, a spectrum of autoinflammatory disorders arising from NLRP3 mutations encoding an aberrant cryopyrin protein and dysregulating the inflammasome.^10^ The proof-of-concept study reported here explored the potential utility of rilonacept in patients with chronic, inflamed joints in whom standard gout treatments were either contraindicated or failed to alleviate pain and inflammation.

## Patients and methods

### Study objectives

The primary objective of this proof-of-concept study was to assess the safety of rilonacept in patients with chronic active gouty arthritis. Secondary objectives were to compare changes in self-reported pain scores, in patients’ and physicians’ global assessments and in levels of high-sensitivity C-reactive protein (hsCRP) during a placebo run-in phase with those during active treatment. Changes in inflammatory pathologies of affected joints with rilonacept treatment were evaluated using a physician-assessment tool designed specifically for this study that takes into account both symptoms and severity in affected joints.

### Study design

The study was reviewed and approved by local institutional review boards, consistent with good clinical practice and applicable regulatory requirements. Written consent was obtained from all patients. In this 14-week, multicentre, non-randomised, monosequence crossover study, a 1-week screening period was followed by a single-blind placebo run-in period of 2-weeks’ duration (week 0 through the end of week 1). Subsequently, patients entered the active treatment period (week 2 through the end of week 7). A 6-week rilonacept withdrawal period (week 8 through the end of week 13) completed the study.

### Study population

Major inclusion criteria were diagnosis by a doctor of chronic active monoarticular or polyarticular gouty arthritis for at least 6 months, with one or more continuously inflamed joints (self-reported or otherwise) for the 4 or more weeks before screening. Patients had pain scores of ⩾3 on a 0–10 point visual analogue scale (VAS). Diagnosis of gout was based on detection of MSU crystals in the synovial fluid, chronically raised serum urate levels and/or tophi. Standard treatments for gout, hyperuricaemia, flare prophylaxis, or pain had been ineffective or involved risks related to side effects. Patients were men and women at least 18 years of age; women of reproductive age agreed to meet contraception requirements.

Among reasons for exclusion were chronic or active infection (systemic or joint); estimated glomerular filtration rate <30 ml/min; treatment with a live (attenuated) virus vaccine during the 3 months before study entry; treatment (<5 half-lives) with an IL1 or a tumour necrosis factor inhibitor; and a history of listeriosis or tuberculosis.

### Treatment

**Figure 1:**
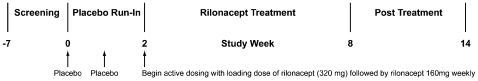
Study design schematic.

After eligibility determinations, patients were screened within 7 days of the start of the study. Day 0 (baseline) to the end of week 1 was the single-blind placebo run-in period. The active treatment period began at week 2 when all patients were switched to receive single-blind rilonacept, beginning with a loading dose of 320 mg (two 2 ml injections) administered subcutaneously, followed by rilonacept 160 mg once a week for weeks 3 through 7 and ended with the post-treatment withdrawal phase beginning at week 8. This dosing regimen has been shown to be well tolerated and efficacious in patients with cryopyrin-associated periodic syndromes, including improvement in joint pains, fever and rash,^11^ and was felt to offer the possibility of a favourable risk–benefit assessment. Figure 1 presents the study design, including dosing and assessment schedule.

Patients were allowed to continue taking their previously prescribed drugs, including aspirin (⩽325 mg/day), NSAIDs (to remain at a stable dose during the trial), allopurinol (if taken for at least 2 months before baseline), probenecid and colchicine (⩽1.2 mg daily, if taken at a stable dose for at least 1 month before baseline).

### Safety assessments

Safety was assessed by monitoring treatment-emergent adverse events (AEs); laboratory values, including anti-rilonacept antibodies; vital signs; chest *x*-ray findings; electrocardiograms; physical examinations; and concomitant drugs. AEs and serious AEs were collected from the time the subject signed the informed consent form through the final study visit.

### Efficacy assessments

Primary efficacy was determined by patient’s pain scores on a 10-point/21-increment VAS which were determined at each visit for the preceding 24 h. Patients were asked to indicate the level of pain they had experienced by filling in a circle on the scale, where “0” was representative of “no pain,” and “10” was representative of “severe pain.” Additional efficacy end points included patients’ and physicians’ global assessments; percentage of responders with 50% and 75% improvements in VAS pain scores; and hsCRP levels. To specifically examine joint pathologies, the following mean changes were assessed: (*a*) the number of all affected joints in which at least one of three symptoms or signs (swelling, tenderness, or erythema) were present (1 point/joint); (*b*) symptom-adjusted score (number of symptoms/joint (maximum: 3 points/joint); and (*c*) symptom severity-adjusted score, weighting by severity of each symptom (1, mild; 2, moderate; 3, severe) (maximum: 9 points/joint if the joint was severely swollen, severely tender and severely erythematous).

### Data analysis and statistics

Three comparisons were made for each variable assessed: run-in phase or placebo effect, by change from baseline to week 2; active-treatment effect, by change from end of week 2 to end of week 8; and post-treatment phase or withdrawal effect, by change from week 8 to week 14. Continuous variables were analysed using the signed-rank test. Proportions were analysed by comparing the proportion with 10% using a binomial test.

## Results

Of the 15 patients screened, 10 were enrolled and treated. One patient withdrew owing to an AE. Table 1 lists the demographic characteristics of the 10 patients treated.

**Table 1:** Patient (n = 10) demographics and baseline characteristics*

Baseline characteristics	Value
Age at screening (years)	
Mean (SD)	61.5 (10.0)
Median	59.5
Minimum, maximum	50, 78
	
Sex, n (%)	
Male	8 (80)
Female	2 (20)
	
Race, n (%)	
Black or African-American	1 (10)
White	9 (90)
	
Ethnicity, n (%)	
Non-Hispanic/Latino	10 (100)
	
Comorbidities at baseline, n (%)	
Hypertension	10 (100)
Obesity	8 (80)
Hyperlipidaemia	5 (50)
Other cardiac disorders (CHF, CAD)	3 (30)
Upper gastrointestinal disorders	2 (20)
Depression	2 (20)
Anxiety	2 (20)
Moderate renal disease†	2 (20)
Diabetes	1 (10)
	
Duration of gout (years)	
Mean	13
Minimum, maximum	3, 26
	
Tophi in ⩾1 joint, n (%)	5 (50)
	
Weight (kg)	
Mean (SD)	105.3 (20.0)
Median	107.6
Minimum, maximum	62.6, 131.5
	
Height (cm)	
Mean (SD)	171.2 (10.6)
Median	170.2
Minimum, maximum	156.3, 187.2

*All patients treated; †glomerular filtration rate (GFR) 30–59 ml/min; patients with GFR <30 ml/min were excluded from the study.

CAD, coronary artery disease; CHF, congestive heart failure.

### Safety

All patients enrolled received all protocol-specified doses of rilonacept, except for one patient who discontinued treatment early. One treated patient, a 50-year-old man, withdrew because of an adverse event after the second week of rilonacept injections owing to severe injection site erythema and induration, both judged to be related to study treatment by the investigator and both resolved without sequelae.

AEs with rilonacept were rare and most often involved the injection site. No deaths and no serious AEs occurred during the study. Vital signs, urine analysis, chemistry, or other laboratory parameters remained without clinically significant changes.

Three patients in this study tested positive for anti-rilonacept antibodies at some time point. All antibody titres were low (⩽1/200) or equal to the minimum required dilution (1/100). The activity of these antibodies was not rilonacept neutralising. No correlation was apparent with any specific AE in patients with anti-rilonacept antibodies and did not appear to affect efficacy.

### Efficacy

**Figure 2:**
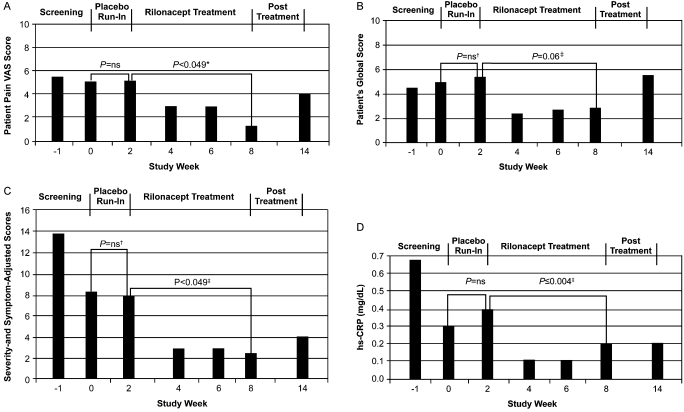
(A) Patient pain visual analogue score (VAS) (median). *p Value from signed-rank test. Pain scores indicate the level of pain experienced by the patient over the preceding 24 h and reported at a study visit. On a 10-point scale, “0” represented “no pain,” and “10” represented “severe pain.” (last observation carried forward (LOCF); n = 10). The LOCF was used to assign any missing values. (B) Patient’s global assessment score (median). On a 10-point scale, “0” represented normal/none and “10” represented severe; LOCF; n = 10. ^†^Week 2 vs day 0; ^‡^week 8 vs week 2 (see online supplementary text files 1 and 2). (C) Symptom and severity-adjusted joint scores (median). Symptom-severity adjusted joint scores were derived by weighting the joint count by severity (1, mild; 2, moderate; 3, severe) for each symptom (swelling, tenderness and erythema) for each joint for a possible maximum score of 9 per joint. ^†^Week 2 vs day 0; ^‡^week 8 vs week 2. (D) High-sensitivity C-reactive protein (hsCRP) levels (median). Normal limit <0.287 mg/dl (defined by central laboratory). ^†^Week 2 vs day 0; ^‡^week 8 vs week 2.

During the first 2 weeks of the active treatment phase, median patients’ self-reported VAS pain scores were significantly reduced from the placebo run-in phase (5.0 to 2.8; p<0.049) with sustained improvement after 6 weeks of rilonacept treatment (week 8) (1.3; p<0.049) (fig 2A). Patients’ (fig 2B) and physicians’ median global assessment scores showed a trend towards “feeling well” from the placebo run-in to the end of active treatment (week 8) and shifted back towards “feeling very unwell” during the post-treatment follow-up phase.

This short-term treatment with rilonacept had no significant effect on the number of affected joints (online supplementary fig 1). However, when the number and severity of symptoms in all joints were considered, significant differences were seen with rilonacept treatment. The median symptom-adjusted joint scores fell from 7.0 at week 2 (end of placebo run-in) to 2.0 with 6 weeks of treatment (week 8) (p<0.049) (online supplementary fig 2), while the symptom severity-adjusted median score for all affected joints also fell significantly, from 8.0 at week 2 baseline to 2.5 at week 8 (p<0.049) (fig 2C).

The median symptom-adjusted joint scores and severity-adjusted joint scores were 2.0 and 2.5 at week 8 (end of treatment) and 2.0 and 4.0 at week 14 (after withdrawal of treatment), respectively (online supplementary fig 2 and fig 2C).

### Changes in CRP

Median hsCRP levels were significantly decreased with rilonacept treatment from 0.4 mg/dl at week 2 (end of placebo run-in) to 0.1, 0.1 and 0.2 mg/dl (p = 0.002, 0.010 and 0.004) at weeks 4, 6 and 8, respectively (normal level <0.3 mg/dl) (fig 2D).

### Responder analysis

**Figure 3:**
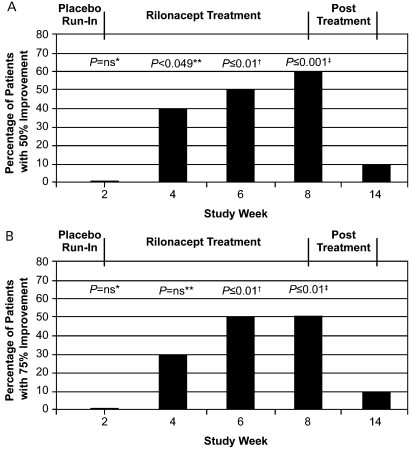
(A) Pain responder analysis. Last observation carried forward (LOCF) (n = 10). (B) Pain responder analysis. LOCF (n = 10). *Week 2 to day 0; **week 4 vs week 2; ^†^week 6 vs week 2; ^‡^week 8 vs week 2.

After 6 weeks of rilonacept treatment, 6/of 10 (60%) patients reported a percentage change in self-reported pain scores that represented at least a 50% improvement (p<0.001); 5/10 (50%) of patients had at least a 75% improvement with rilonacept (p⩽0.01) (fig 3).

## Discussion

This pilot study explored the potential utility of inhibition of IL1 in patients with chronic active gout, assessing the safety profile of rilonacept and evaluating changes in disease activity with its use. The study design, which was monosequence crossover and non-randomised, including a single-blind placebo run-in followed by active treatment and subsequent withdrawal period, allowed for this assessment. In this patient population, rilonacept was generally well tolerated. Injection site reactions were the most common AE, usually mild in severity.

Rilonacept administration for 6 weeks also yielded clinical improvements; specifically, a trend towards progressive improvement over time. Patients’ self-reported median pain VAS scores steadily and significantly decreased over the course of rilonacept treatment. The majority of patients reported at least a 75% improvement in pain after rilonacept treatment that was not seen after withdrawal of rilonacept; hsCRP levels significantly decreased with treatment. This acute phase reactant change is consistent with evidence correlating circulating levels of CRP with acute gouty attacks.^12^ Although preliminary, and using a non-validated instrument, symptom-adjusted and severity-adjusted joint scores showed significant improvements with rilonacept treatment. These adjusted scores provided a means to detect significant changes in joint pathologies with treatment.

Consistent with results in a recent safety and pharmacokinetics trial involving patients with end-stage renal disease,^13^ the dosing of rilonacept in this study was not adjusted in the two patients with moderately impaired kidney function.

The primary limitations of this study were the small cohort size, a short placebo run-in phase and the absence of independent blinded joint assessors. This study also did not directly assess the relative contributions to chronic gouty arthritis of IL1α and IL1β, both of which are inhibited by rilonacept. Despite these limitations, this study demonstrated statistically significant improvement in clinical and laboratory measures of gout.

In conclusion, this proof-of-concept study provided a positive signal to suggest that rilonacept may offer a well-tolerated approach for reducing pain in patients with chronic active difficult gouty arthritis not adequately managed with other treatments. The results further support the hypothesis that IL1 blockade may represent a useful and selective treatment strategy for the growing population of patients with gout, including chronic, refractory gouty arthritis, suggesting that rilonacept should be studied further in patients with gout.

## References

[1] Edwards NL Treatment-failure gout: A moving target. Arthritis Rheum 2008;58:2587–90 18759307 10.1002/art.23803

[2] Terkeltaub RA Clinical practice. Gout. N Engl J Med 2003;349:1647–55 14573737 10.1056/NEJMcp030733

[3] Sundy JS Becker MA Baraf HS Reduction of plasma urate levels following treatment with multiple doses of pegloticase (polyethylene glycol-conjugated uricase) in patients with treatment-failure gout: results of a phase II randomized study. Arthritis Rheum 2008;58:2882–91 18759308 10.1002/art.23810

[4] Becker MA Schumacher HR Benjamin KL Quality of life and disability in patients with treatment-failure gout. J Rheumatol 2009;36:1041–8 19332629 10.3899/jrheum.071229

[5] Taylor WJ Schumacher HR Jr Singh JA Assesment of outcome in clinical trials of gout – a review of current measures. Rheumatology (Oxford) 2007;46:1751–76 17650521 10.1093/rheumatology/kem178

[6] Pope RM, Tschopp The role of interleukin-1 and the inflammasome in gout: implications for therapy. Arthritis Rheum 2007;56:3183–8 17907163 10.1002/art.22938

[7] Martinon F Petrilli V Mayor A Gout-associated uric acid crystals activate the NALP3 inflammasome. Nature 2006;440:237–41 16407889 10.1038/nature04516

[8] Cronstein RN Terkeltaub R The inflammatory process of gout and its treatment. Arthritis Res Ther 2006;8 (Suppl 1):S3 16820042 10.1186/ar1908 PMC3226108

[9] So A De Smedt T Revaz S A pilot study of IL-1 inhibition by anakinra in acute gout. Arthritis Res Ther 2007;9:R28 17352828 10.1186/ar2143 PMC1906806

[10] Aksentijevich I Putnam CD Remmers EF The clinical continuum of cryopyrinopathies: novel CIAS1 mutations in North American patients and a new cryopyrin model. Arthritis Rheum 2007;56:1273–85 17393462 10.1002/art.22491 PMC4321998

[11] Hoffman HM Throne ML Amar NJ Efficacy and safety of rilonacept (IL-1 trap) in cryopyrin-associated periodic syndromes (CAPS): results from two sequential placebo-controlled studies. Arthritis Rheum 2008;58:2443–52 18668535 10.1002/art.23687

[12] Urano W Yamanaka H Tsutani H The inflammatory process in the mechanism of decreased serum uric acid concentrations during acute gouty arthritis. J Rheumatol 2002;29:1950–3 12233891

[13] Radin A Marbury T Osgood G Safety and pharmacokinetics of subcutaneously administered rilonacept in subjects with well-controlled end-stage renal disease (ESRD). J Clin Pharmacol (in press). 10.1177/0091270009351882 20035038

